# Neonatal dexamethasone treatment exacerbates hypoxic-ischemic brain injury

**DOI:** 10.1186/1756-6606-6-18

**Published:** 2013-04-18

**Authors:** Kan-Hsun Chang, Che-Ming Yeh, Chia-Yu Yeh, Chiung-Chun Huang, Kuei-Sen Hsu

**Affiliations:** 1 Department of Pharmacology, College of sMedicine, National Cheng Kung University, Tainan, 701, Taiwan; 2 Institute of Basic Medical Sciences, College of Medicine, National Cheng Kung University, Tainan, 701, Taiwan

## Abstract

**Background:**

The synthetic glucocorticoid dexamethasone (DEX) is commonly used to prevent chronic lung disease in prematurely born infants. Treatment regimens usually consist of high doses of DEX for several weeks, notably during a critical period of brain development. Therefore, there is some concern about adverse effects of this clinical practice on fetal brain development. In this study, using a clinically relevant rat model, we examined the impact of neonatal DEX treatment on subsequent brain injury due to an episode of cerebral hypoxia-ischemia (HI).

**Results:**

We found that a 3-day tapering course (0.5, 0.3 and 0.1 mg/kg) of DEX treatment in rat pups on postnatal days 1–3 (P1-3) exacerbated HI-induced brain injury on P7 by a glucocorticoid receptor-mediated mechanism. The aggravating effect of neonatal DEX treatment on HI-induced brain injury was correlated with decreased glutamate transporter-1 (GLT-1)-mediated glutamate reuptake. The expression levels of mRNA and protein of GLT-1 were significantly reduced by neonatal DEX treatment. We also found that the administration of β-lactam antibiotic ceftriaxone increased GLT-1 protein expression and significantly reduced HI-induced brain injury in neonatal DEX-treated rats.

**Conclusions:**

These results suggest that early DEX exposure may lead the neonatal brain to be more vulnerable to subsequent HI injury, which can be ameliorated by administrating ceftriaxone.

## Background

Chronic lung disease (CLD) is an important cause of mortality and morbidity in preterm infants and inflammation plays a major role in its pathogenesis [1,2]. Due to their strong anti-inflammatory properties, synthetic glucocorticoids such as dexamethasone (DEX) or betamethasone are frequently used to prevent or lessen the morbidity of CLD in preterm infants. Given that the brain is a major target for glucocorticoids and the developing brain is inherently more susceptible to drug-induced alterations than the adult brain [3], there is concern that neonatal DEX therapy may be associated with increased risk of adverse neurologic outcomes in later life. While there are some controversies about its adverse effects on neurodevelopment, numerous clinical studies have demonstrated that premature infants receiving DEX therapy have a higher incidence of neuromotor dysfunction and an increased risk of developing cerebral palsy (CP) [4-7]. There is also experimental evidence that DEX exposure in the neonatal rat pups can lead to alterations in hippocampal synaptic plasticity and deficits in learning and memory [8-10]. Although these results highlight the adverse consequences of neonatal DEX treatment on brain development, little is known about the molecular mechanisms behind these abnormalities.

Neonatal hypoxia-ischemia (HI) is a leading cause of perinatal brain injury, which may ultimately lead to CP, mental retardation, learning disability and epilepsy [11]. Using different neonatal rat models of HI, previous studies have revealed that HI-induced brain injury is associated with excitotoxicity, a type of neuronal death triggered by overstimulation of glutamate receptors and loss of calcium homeostasis [12,13]. Interestingly, there is evidence that pretreatment of neonatal rats with DEX prevents brain injury associated with cerebral HI [14-16]. These findings contrast with clinical observations that early DEX administration in preterm infants may increase the incidence of CP [4-6]. These seemingly discrepant findings are likely related to variations in timing and dosage regimens. It was deemed of interest to perform a detailed analysis of the influence of neonatal DEX treatment on subsequent HI-induced brain injury by using a protocol resembling the one used in clinical practice for preterm infants. In this study, using a well established and clinically relevant 3-day tapering course of DEX treatment in neonatal rat pups on postnatal days 1–3 (P1-3) [9,14,17,18], we asked two main questions: (1) whether neonatal DEX treatment alters the vulnerability of the immature brain to HI-induced brain injury and (2) if so, what is the responsible molecular mechanism(s).

## Results

### Neonatal DEX treatment enhances HI-induced brain injury

**Figure 1:**
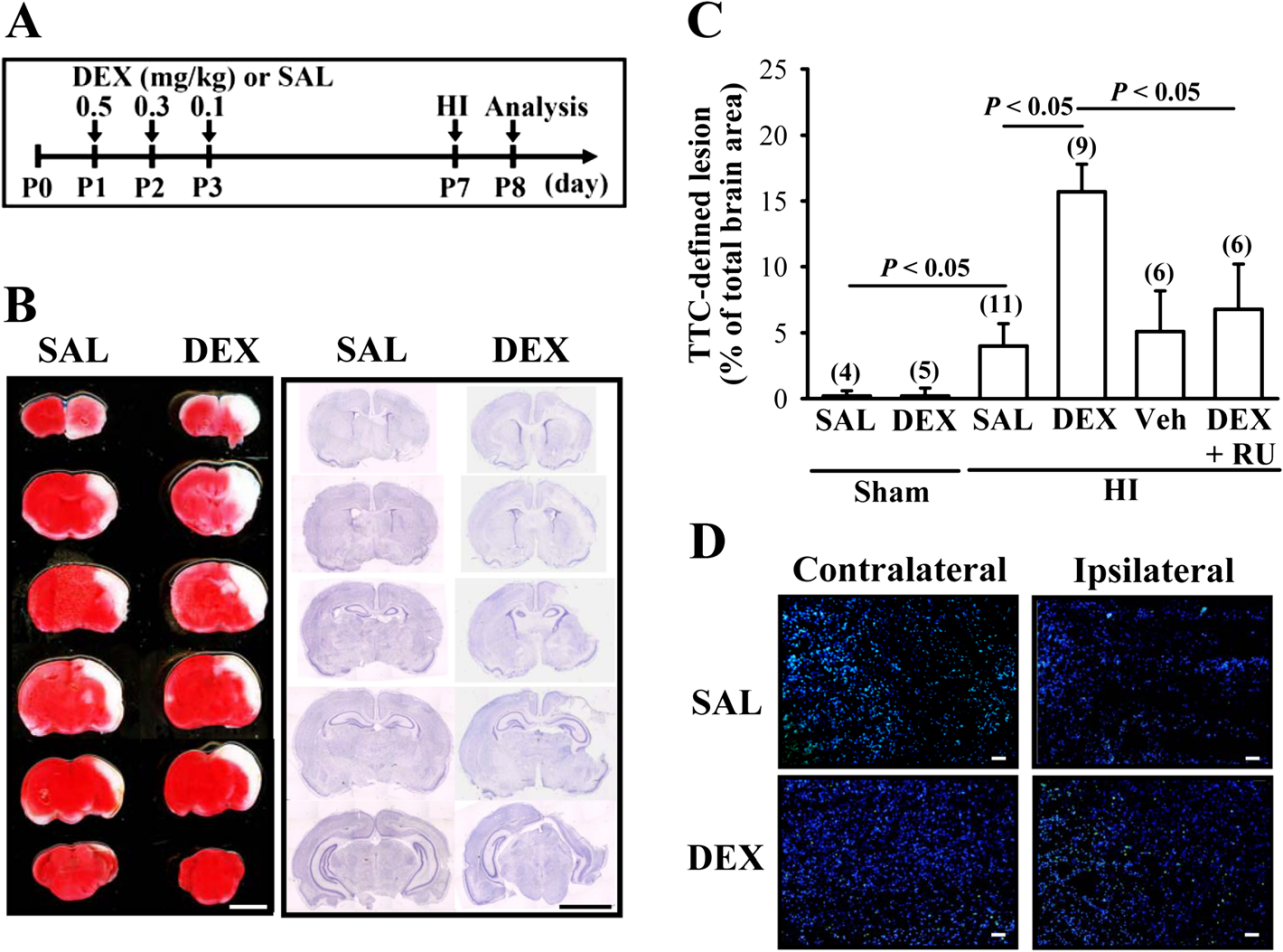
**Effect of neonatal DEX treatment on HI-induced brain injury.** (**A**) Schematic representation of the experimental design for examining the effect of neonatal DEX treatment on subsequent HI-induced brain injury. Rat pups were treated with a 3-day tapering course (0.5, 0.3 and 0.1 mg/kg) of DEX or equal volume of SAL from P1 to P3 and were subjected to cerebral HI by unilateral ligation of the right common carotid artery followed by exposure to hypoxia (92% N_2_ + 8% O_2_) for 2 hours on P7. Brain injury was analyzed on P8. (**B**) TTC (left column) and Nissl staining (right column) of serial coronal sections 24 hours after HI. DEX-treated group exhibited more marked infarctions (white areas), particularly in the ipsilateral cerebral cortex and the hippocampus, than SAL-treated group. Scale bar is 0.5 cm. (**C**) Brain injury was quantified at 24 hours after HI by TTC staining. Sham denotes animals received right carotid artery exposure without ligation and were exposed to normoxic condition. (**D**) Fluorescent TUNEL staining (TUNEL-positive cells are green) with DAPI counterstain (blue) was utilized to visualize TUNEL labeling of apoptotic cells in the frontal cortex of neonatal SAL- or DEX-treated rat pups 24 hours after HI. Similar results were observed in another four rat pups in each group. Scale bar is 100 μm. The numbers in *parentheses* indicate the number of animals examined. Data are presented as means ± S.E. M.

To determine the influence of neonatal DEX treatment on HI-induced brain injury, we compared the infarction areas in coronal sections of SAL and DEX groups 24 hours after experimental HI (Figure 1A). We chose this time point because it has been reported to be the peak in expression of neonatal HI-induced cell damage [19]. Figure 1B shows representative images from HI rats stained with 2,3,5-triphenyltetrazolium chloride (TTC) or cresyl violet. Neonatal DEX-treated group exhibited more severe HI-induced brain injury, particularly in the cerebral cortex and the hippocampus, than SAL-treated group. One-way ANOVA revealed a significant main effect of HI treatment on infarct volume (F_3,25_ = 15.3, *P* < 0.001), and post hoc analysis showed that infarct volume was significantly increased (*P* < 0.05) by neonatal DEX treatment compared with SAL-treated group (Figure 1C). The enhancement effect of neonatal DEX treatment on HI-induced brain injury was prevented when the animals were given glucocorticoid receptor (GR) antagonist, RU 38486 (40 mg/kg), 1 hour before daily DEX treatment (*P* < 0.05 vs. DEX). TUNEL analysis was used to determine whether neonatal DEX treatment may sensitize HI-induced cell damage. As shown in Figure 1D, TUNEL-positive apoptotic cells were evident within the frontal cortex ipsilateral to common carotid artery ligation 24 hours after HI. The number of TUNEL-positive cells was significantly greater in DEX-treated group than saline-treated group.

### Neonatal DEX treatment decreases GLT-1-mediated glutamate reuptake

**Figure 2:**
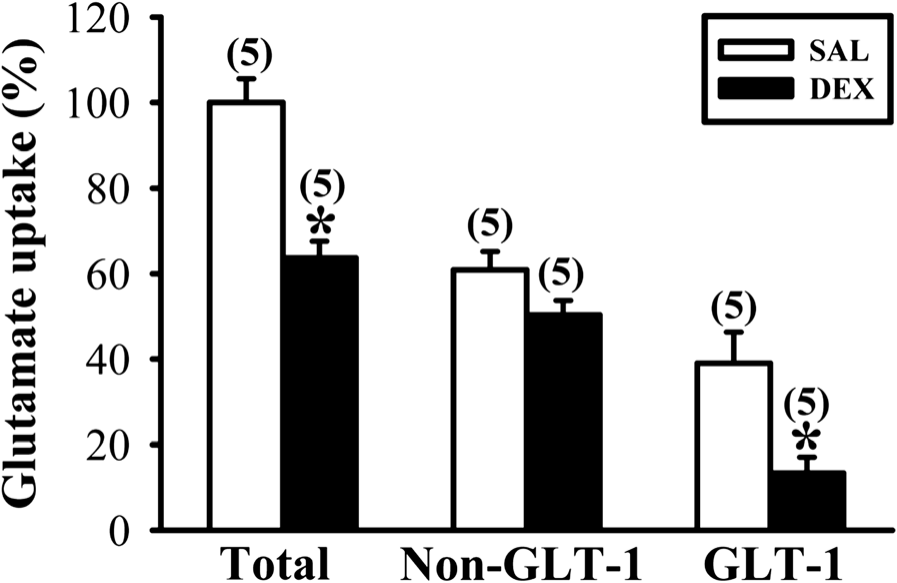
**Effect of neonatal DEX treatment on glutamate uptake activity in gliosomes.** Summary bar graphs depicting glutamate uptake activity in gliasomes from the frontal cortex of SAL- and DEX-treated groups on P7. DEX-treated group exhibited a significant decrease of total, and GLT-1-mediated glutamate uptake compared with those of SAL-treated group. The non-GLT-1-mediated glutamate uptake was calculated in the presence of GLT-1 inhibitor dihydrokainate (100 μM). The numbers in parentheses indicate the number of animals examined. Data are presented as means ± S.E.M. *P < 0.05 compared with SAL-treated group.

Because excitotoxicity due to excessive extracellular glutamate is closely associated with HI-induced brain injury [12,13] and transporter-mediated glutamate uptake is essential for maintaining low extracellular glutamate concentrations [20], we therefore examined the influence of neonatal DEX treatment on basal glutamate uptake activity in gliosomes from the frontal cortex on P7. The amounts of total and GLT-1-mediated glutamate uptake in gliosomes were significantly reduced in DEX-treated group compared with SAL-treated group (Total: F_1,8_ = 28.3; *P* < 0.001; GLT-1: F_1,8_ = 9.8; *P* = 0.014; Figure 2). Although the amount of non-GLT-1-mediated glutamate uptake tended to be lower in DEX-treated group compared with that from SAL-treated group, the difference did not reach statistical significance (F_1,8_ = 3.7; *P* = 0.08). In addition, the observed reduction in basal glutamate uptake activity in DEX-treated group was prevented when rat pups were given RU 38486 (40 mg/kg) 1 hour before daily DEX treatment (F_1,14_ = 21.7; *P* < 0.001; data not shown).

### Neonatal DEX treatment decreases basal GLT-1 and GLAST mRNA and protein expression

**Figure 3:**
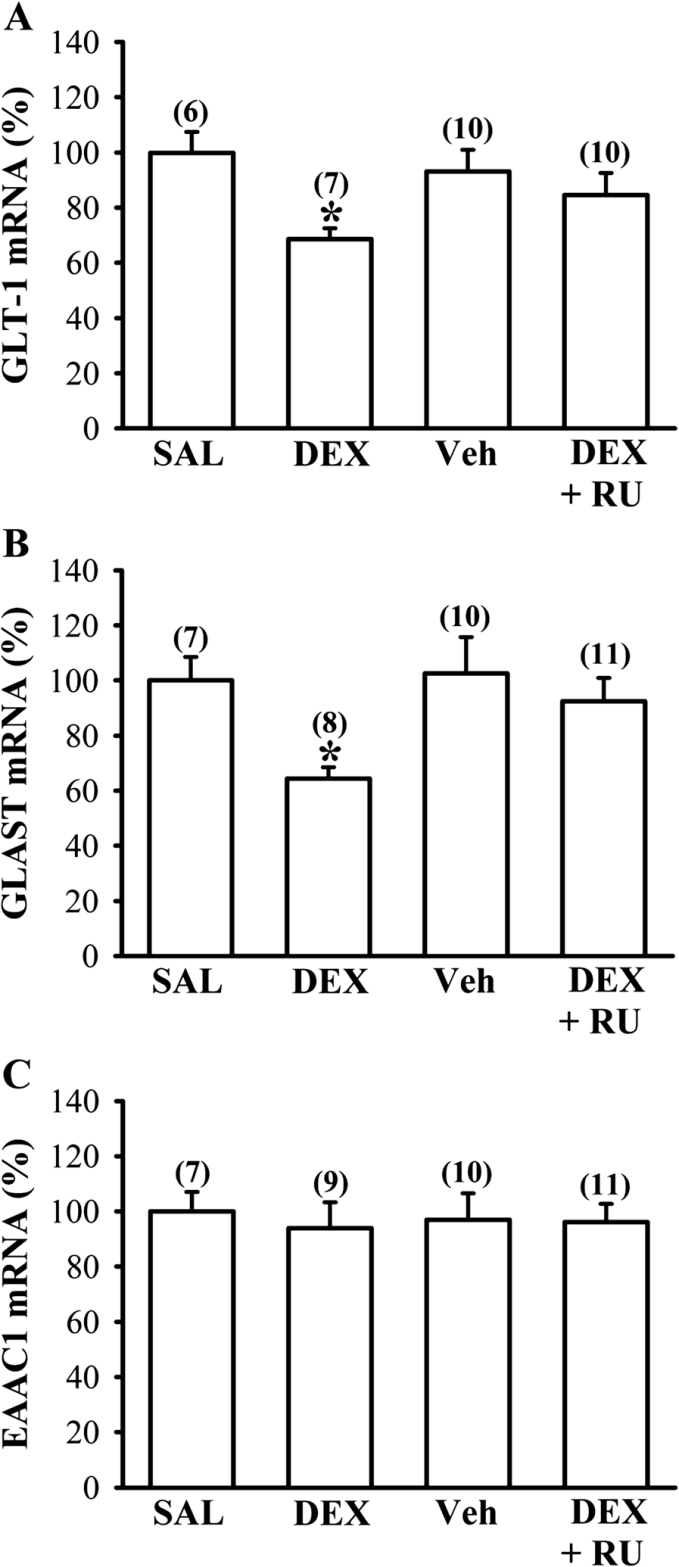
**Effect of neonatal DEX treatment on the expression of glutamate uptake transporter mRNAs in the frontal cortex.** (**A-C**) Quantitative real-time PCR analysis showing the relative expression levels of GLT-1 (**A**), GLAST (**B**), and EAAC1 (**C**) mRNA in the frontal cortex from SAL-, DEX-, vehicle (Veh)- and DEX + RU 38486 (RU)-treated groups on P7. The vehicle group received equivalent volume of intraperitoneal injection of propylene glycol. The numbers in *parentheses* indicate the number of animals examined. Data are presented as means ± S.E.M. *P < 0.05 compared with SAL-treated group.

**Figure 4:**
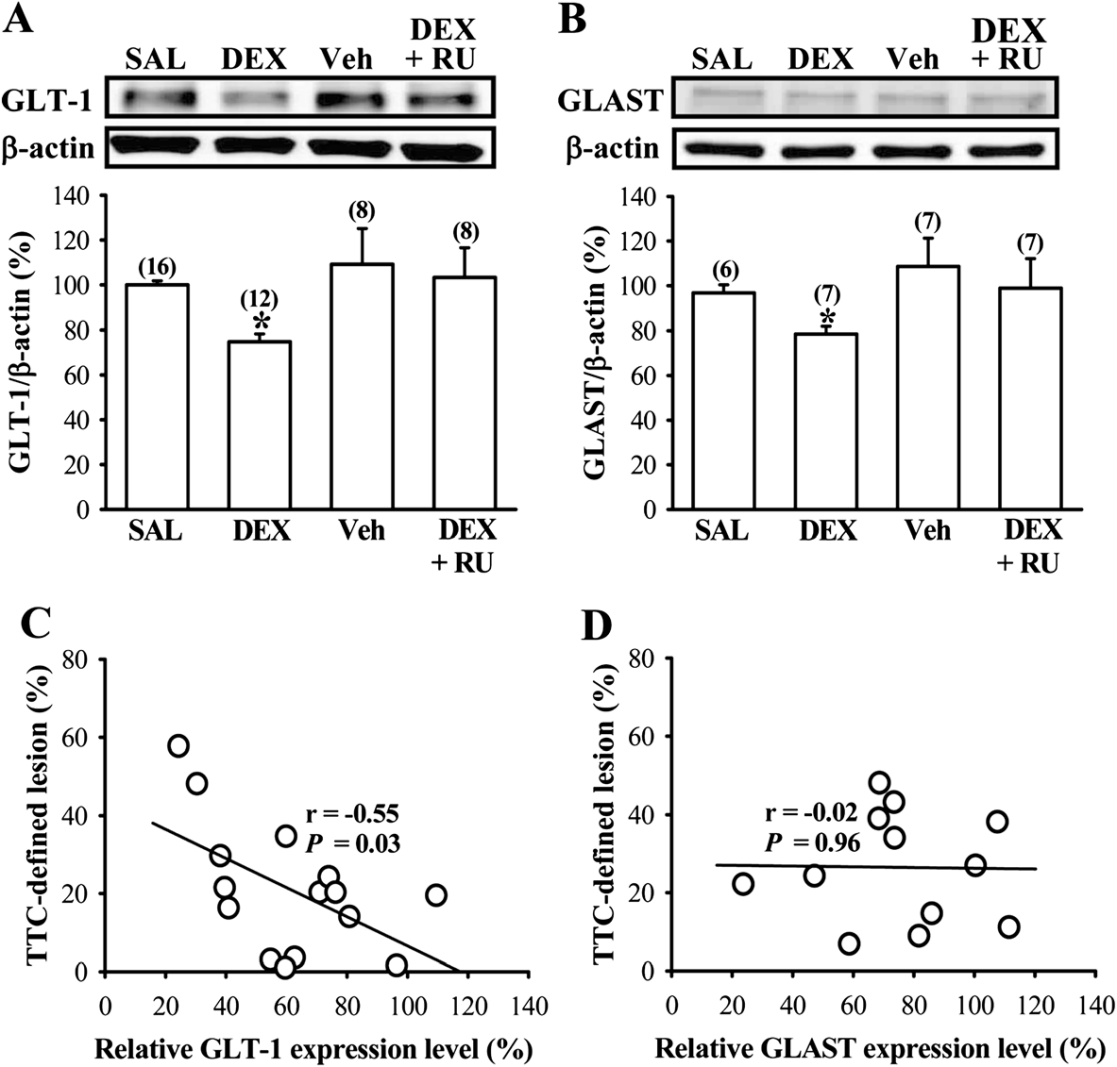
**Effect of neonatal DEX treatment on the expression of glutamate uptake transporter proteins in the frontal cortex.** (**A** and **B**) Representative Western blot and summary bar graph depicting the relative expression levels of GLT-1 (**A**) and GLAST protein (**B**) in the frontal cortex from SAL-, DEX-, vehicle (Veh)- and DEX + RU 38486 (RU)-treated groups on P7. (**C**) A significant inverse correlation was found between the extent of TTC-defined infarct volume 24 hours after HI and the relative levels of GLT-1 protein. (**D**) The extent of TTC-defined infarct volume 24 hours after HI was not correlated with the relative levels of GLAST protein. The numbers in parentheses indicate the number of animals examined. Data are presented as means ± S.E.M. *P < 0.05 compared with SAL-treated group.

So far, five distinct mammalian glutamate transporter subtypes, termed EAAT1 (glutamate-aspartate transporter, GLAST), EAAT2 (GLT-1), EAAT3 (excitatory amino acid carrier 1, EAAC1), EAAT4 and EAAT5, have been cloned. GLAST and GLT-1 are found predominantly in glial cells and EAAC1 is expressed in both neurons and glial cells [21]. Because the observed reduction of glutamate uptake in DEX-treated group could be a result of decreased expression of glutamate transporters, we therefore examined the influence of neonatal DEX treatment on the expression of basal glutamate transporters in the frontal cortex on P7. Quantitative real-time RT-PCR analysis showed that neonatal DEX treatment led to decreased expression of GLT-1 (F_1,11_ = 14.7; *P* < 0.01) and GLAST mRNAs (F_1,13_ = 15.5; *P* < 0.01) compared with SAL-treated group (Figures 3A and B). The observed reduction in GLT-1 and GLAST mRNA expression in DEX-treated group was prevented when rat pups were given RU 38486 (40 mg/kg) 1 hour before daily DEX treatment (*P* < 0.05 vs. DEX). However, there was no significant difference between DEX- and SAL-treated group in EAAC1 mRNA expression (F_1,14_ = 0.2; *P* = 0.63). In parallel, the levels of GLT-1 and GLAST proteins in the frontal cortex were noted to be decreased in DEX-treated group compared with SAL-treated group on P7 (GLT-1: F_1,26_ = 47.3; *P* < 0.001; GLAST: F_1,11_ = 13.1; *P* < 0.01; Figures 4A and B). The inhibitory effect of neonatal DEX treatment on GLT-1 and GLAST protein expression was prevented when the animals were given RU 38486 (40 mg/kg) 1 hour before daily DEX treatment (*P* < 0.05 vs. DEX).

On the basis of the data demonstrating the downregulation of GLT-1 and GLAST protein levels after neonatal DEX treatment, it was assumed that reduced GLT-1 and GLAST protein levels may contribute to enhanced HI-induced brain injury by neonatal DEX treatment. To test this possibility, we ran a correlation between them in slices prepared from DEX-treated group. A clear inverse correlation was found between the extent of TTC-defined infarct volume 24 hours after HI and the relative levels of GLT-1 protein (*r* = −0.55; *P* = 0.03, n = 15; Figure 4C). In contrast, no such relationship was seen with the relative levels of GLAST protein (*r* = −0.02; *P* = 0.96, n = 12; Figure 4D).

### DEX treatment decreases GLT-1 mRNA and protein expression in C6 glioma cells

**Figure 5:**
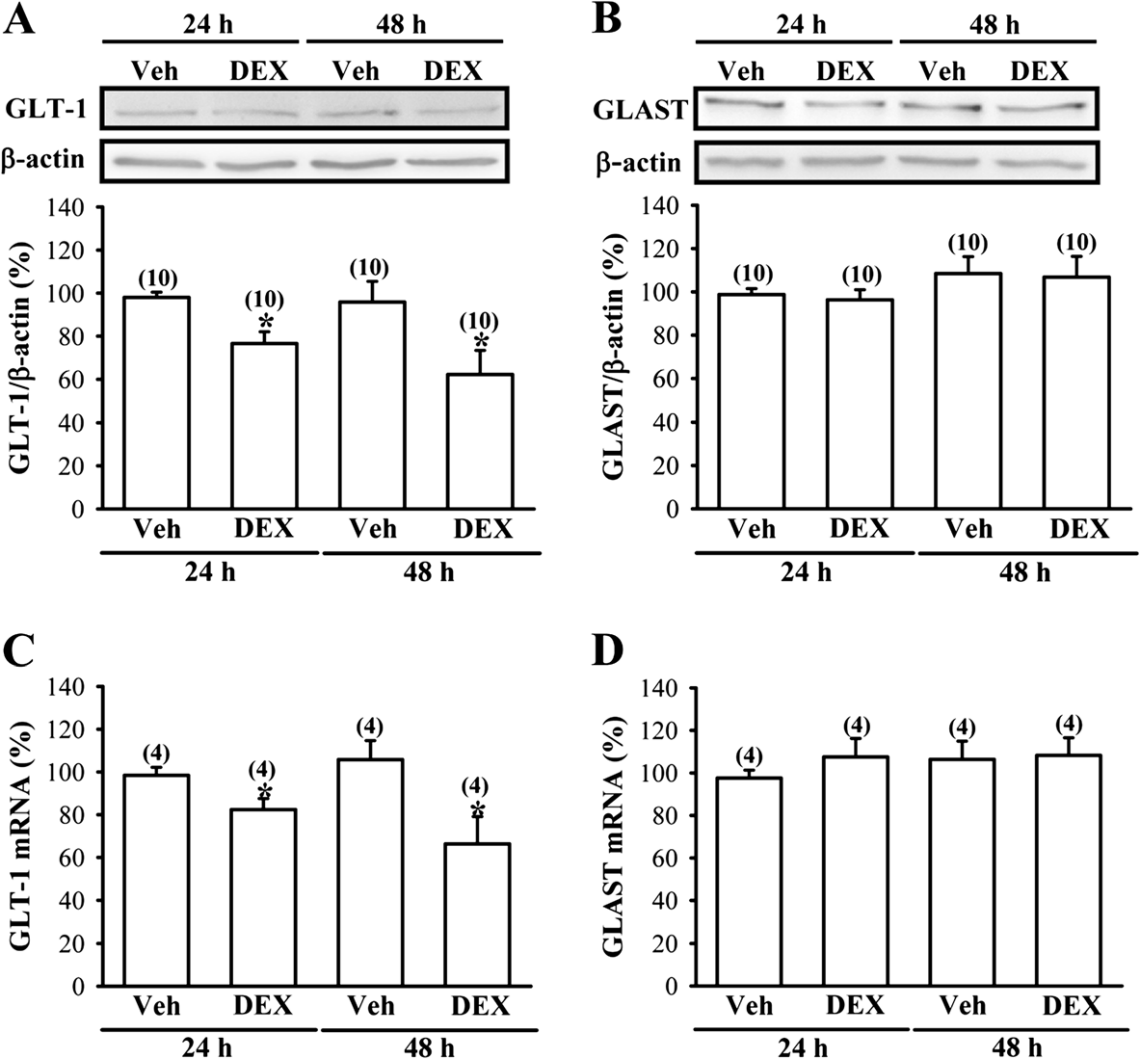
**Effect of DEX treatment on the expression of glutamate uptake transporter proteins and mRNAs in C6 glioma cells.** (**A** and **B**) Representative Western blot and summary bar graph depicting the relative expression levels of GLT-1 (**A**) and GLAST protein (**B**) in C6 glioma cells treated with either vehicle (Veh, 0.1% DMSO) or DEX (100 μM) for 24 or 48 hours. (**C** and **D**) Quantitative real-time PCR analysis showing the relative expression levels of GLT-1 (**C**) or GLAST mRNA (**D**) in C6 glioma cells treated with either Veh or DEX (100 μM) for 24 or 48 hours. The numbers in parentheses indicate the number of animals examined. Data are presented as means ± S.E.M. *P < 0.05 compared with Veh-treated group.

As GLT-1 is expressed predominantly in glial cells [20], further experiments were performed to determine whether DEX treatment may downregulate the expression of GLT-1 in glial cell cultures *in vitro*. To test this possibility, rat C6 glioma cells were treated with DEX (100 μM) for 24 and 48 hours, respectively. As expected, GLT-1 protein expression in C6 glioma cells was significantly downregulated by DEX treatment, based on Western blotting analysis of whole-cell lysates (24 hours: F_1,18_ = 12.8; *P* < 0.01; 48 hours: F_1,18_ = 5.2; *P* < 0.05; Figure 5A). In contrast, GLAST protein expression was not altered by DEX treatment (24 hours: F_1,18_ = 0.2; *P* = 0.67; 48 hours: F_1,18_ = 0.1; *P* = 0.89; Figure 5B). In parallel, a significant decrease in GLT-1 mRNA expression was noted in C6 glioma cells of DEX-treated group compared with vehicle-treated group (24 hours: F_1,18_ = 6.2; *P* < 0.05; 48 hours: F_1,18_ = 6.4; *P* < 0.05; Figure 5C), whereas GLAST mRNA level was not altered by DEX treatment (24 hours: F_1,18_ = 1.1; *P* = 0.31; 48 hours: F_1,18_ = 0.1; *P* = 0.86; Figure 5D).

### The expression of *N*-methyl-D-aspartate receptors is not alter by neonatal DEX treatment

**Figure 6:**
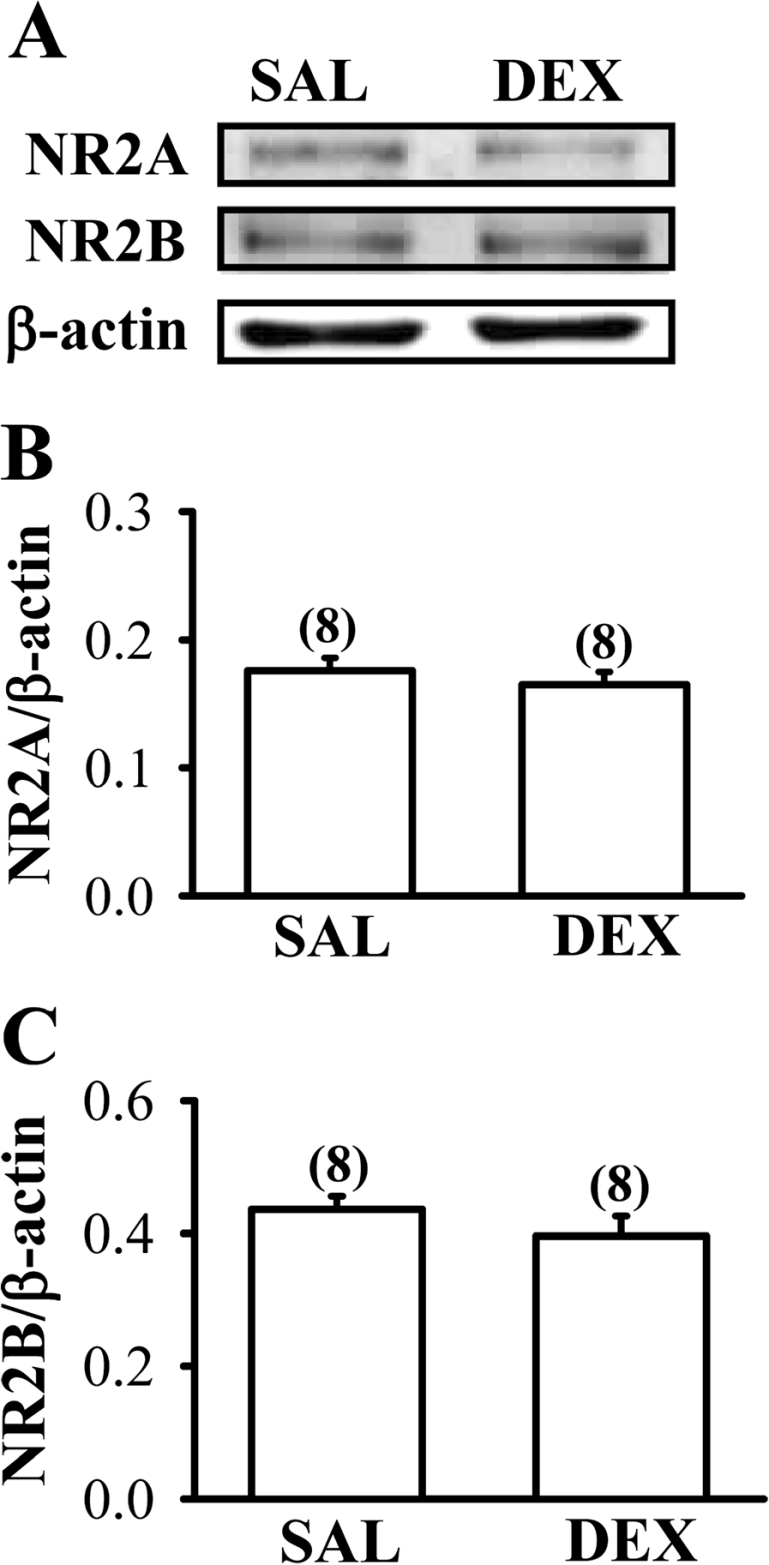
**Effect of neonatal DEX treatment on the expression of NMDA receptor subunit proteins in the frontal cortex.** (**A**-**C**) Representative Western blot (**A**) and summary bar graph depicting the relative expression levels of NR2A (**B**) and NR2B subunit protein (**C**) in the frontal cortex from SAL- and DEX-treated groups on P7. The numbers in parentheses indicate the number of animals examined. Data are presented as means ± S.E.M.

The occurrence of HI-induced excitotoxicity is generally thought to be associated with overstimulation of glutamate receptors, particularly the *N*-methyl-D-aspartate (NMDA) receptor subtype [12,13]. Hence, we determined whether the expression levels of NMDA receptor in the frontal cortex were altered by neonatal DEX treatment. As shown in Figures 6A-C, there were no significant differences between DEX- and SAL-treated group in the expression levels of the two major NMDA receptor subunits, NR2A and NR2B, in the whole tissue lysates of the frontal cortex on P7 (NR2A: F_1,14_ = 0.6; *P* = 0.45; NR2B: F_1,14_ = 1.2; *P* = 0.29).

### Ceftriaxone attenuates HI-induced brain injury in neonatal DEX-treated rat pups

**Figure 7:**
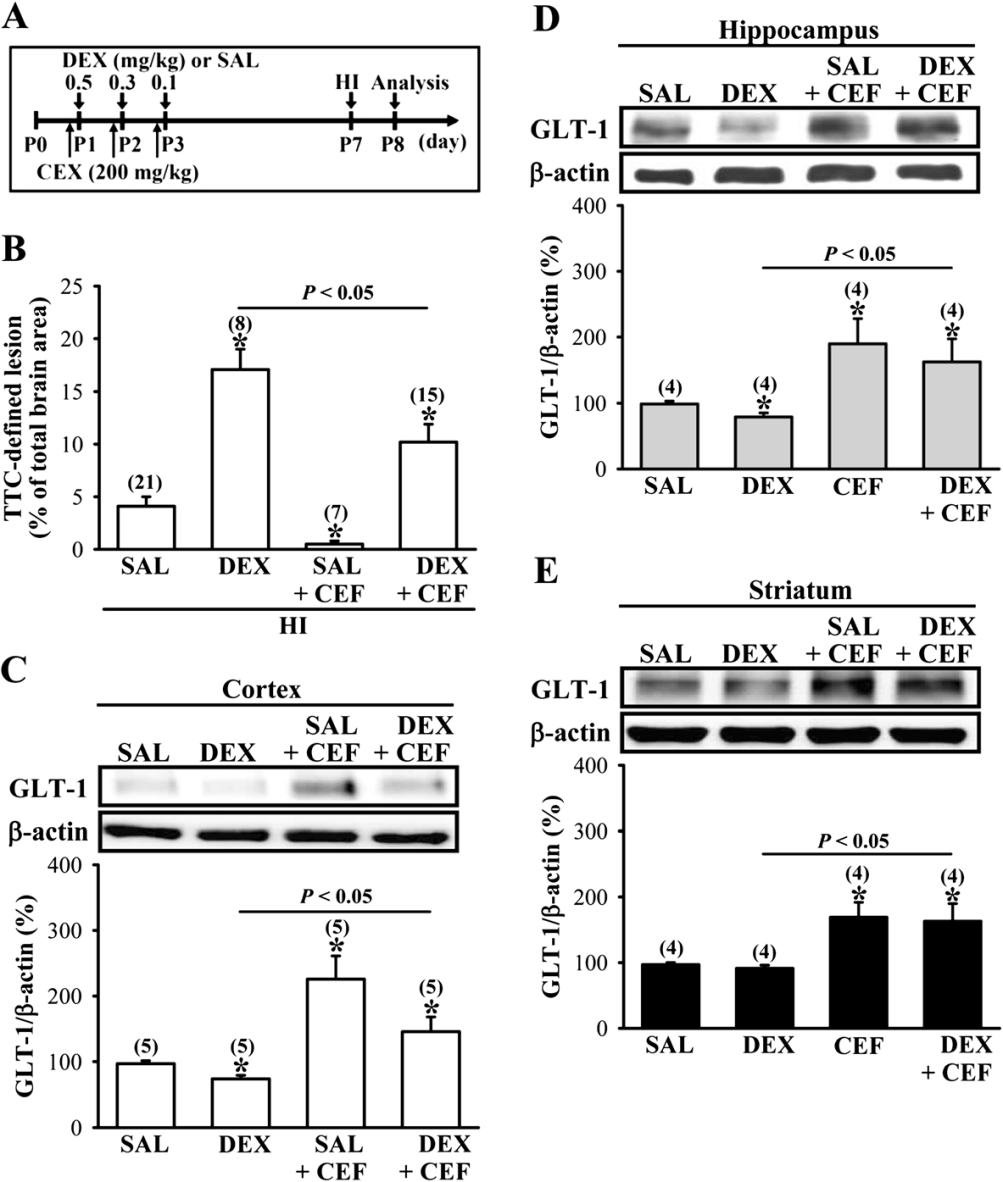
**Ceftriaxone attenuates HI-induced brain injury and the loss of GLT-1 protein in neonatal DEX-treated rats.** (**A**) Schematic representation of the experimental design for examining the effect of ceftriaxone (CEF) treatment on DEX-induced enhancement of HI-induced brain injury. Rat pups were treated with a 3-day tapering course (0.5, 0.3 and 0.1 mg/kg) of DEX or equal volume of SAL from P1 to P3 and were subjected to cerebral HI by unilateral ligation of the common carotid artery followed by exposure to hypoxia (92% N_2_ + 8% O_2_) for 2 hours on P7. Ceftriaxone (200 mg/kg) was injected intraperitoneally 1 hour before DEX or SAL administration. Brain injury and the expression of GLT-1 protein in the frontal cortex were analyzed on P8. (**B**) Brain injury was quantified at 24 hours after HI by TTC staining. (**C**-**E**) Representative Western blot and summary bar graph depicting the relative expression levels of GLT-1 in the frontal cortex (**C**), the hippocampus (**D**) and the striatum (**E**) from SAL-, DEX-, SAL + CEF- and DEX + CEF-treated groups on P7. The numbers in *parentheses* indicate the number of animals examined. Data are presented as means ± S.E.M. *P < 0.05 compared with SAL-treated group.

The above results clearly indicate the importance of GLT-1 levels in determining the vulnerability of the neonatal brain to HI injury. These findings prompted us to investigate whether the elevation of GLT-1 protein expression could attenuate the reinforcing effect of neonatal DEX treatment on HI-induced brain injury. For this, neonatal rat pups were pretreated with β-lactam antibiotic ceftriaxone (200 mg/kg), that have been shown to effectively exert neuroprotective effect against neonatal HI-induced brain injury through the elevation of GLT-1 expression [22], 1 hour before DEX application (Figure 7A). As expected, we observed that ceftriaxone treatment significantly reduced HI-induced brain injury (F_1,26_ = 5.1; *P* < 0.05 vs. SAL) and attenuated the extent of brain damage in DEX-treated rat pups to HI (F_1,21_ = 6.5; *P* < 0.05 vs. DEX; Figure 7B). In addition, ceftriaxone treatment substantially increased GLT-1 protein expression in the frontal cortex (F_1,6_ = 13.1; *P* < 0.01 vs. SAL; Figure 7C), the hippocampus (F_1,6_ = 6.2; *P* < 0.05 vs. SAL; Figure 7D) and the striatum (F_1,6_ = 9.7; *P* < 0.05 vs. SAL; Figure 7E), and completely rescued the inhibitory effect of DEX treatment on GLT-1 protein expression in the frontal cortex (F_1,8_ = 9.6; *P* < 0.05 vs. DEX; Figure 7C) and the hippocampus (F_1,6_ = 7.2; *P* < 0.05 vs. DEX; Figure 7D). In contrast, GLT-1 protein expression in the striatum was not significantly affected by neonatal DEX treatment (F_1,6_ = 0.9; *P* = 0.39 vs. SAL; Figure 7E).

## Discussion

The concern that neonatal DEX therapy might increase risk of developing neurological dysfunction by altering neural development was an important motivation for our study. Using a clinically relevant animal model, our results provide the first evidence that neonatal DEX treatment leads to a sustained downregulation of GLT-1 expression and thereby exacerbates HI-induced brain injury. Moreover, we confirm that ceftriaxone can exert a neuroprotective effect against HI-induced brain injury in neonatal rats by increasing expression of GLT-1 [22] and suggest that pretreatment with ceftriaxone in neonatal rats can effectively attenuate DEX-induced augmentation of HI-induced brain injury.

Excitotoxicity related to extracellular accumulation of glutamate plays a critical role in neonatal HI-induced brain injury [23]. The accumulation of extracellular glutamate may result from either decreased uptake or the reversed action of glutamate transporters [20]. The observation that DEX-treated group showed a significantly lower GLT-1-mediated glutamate uptake activity in gliosomes compared with that from SAL-treated group suggests that neonatal DEX treatment may cause decreased glutamate uptake and result in deleterious enhancement of excitotoxic brain injury to subsequent HI insults. In addition, we observed that neonatal DEX treatment led to a significant decrease in both mRNA and protein levels of GLT-1 and GLAST, indicating that reduced expression of glutamate transporters may account, at least in part, for the decrease in glutamate uptake observed in P7 rat pups. It is however noteworthy that, although expression levels of both GLT-1 and GLAST were downregulated by neonatal DEX treatment, we observed no significant correlation between the extent of HI-induced brain injury and the expression of GLAST protein. In contrast, our results revealed that GLT-1 protein levels are inversely correlated with the extent of HI-induced brain injury. Therefore, we hypothesize that changes in GLT-1 protein expression underlie the enhancement of neonatal HI-induced brain injury found in DEX-treated rats. This hypothesis is further supported by findings that ceftriaxone treatment attenuates the enhancing effect of neonatal DEX treatment on HI-induced brain injury by increasing GLT-1 expression. Moreover, we found no significant differences in expression of NR2A and NR2B subunits in the frontal cortex between DEX- and SAL-treated groups, suggesting that neonatal DEX treatment does not alter the total amount of NMDA receptors.

How does neonatal DEX treatment lead to a lasting decrease in GLT-1 expression? Our finding that administration of RU 38486 almost completely prevented DEX-induced decreases in GLT-1 mRNA and protein expression supports a pathway involving the GR. Although the molecular basis of how GR activation reduces GLT-1 gene transcription remains unclear, it is highly likely that GR acts mainly through interfering with the nuclear transcription factor nuclear factor-κB (NF-κB) signaling to downregulate GLT-1 expression. Using either pharmacological or genetic approaches, the NF-κB signaling has been linked to several drug-induced or neuron-dependent transcriptional activation of GLT-1 [24-26]. Thus, downregulation of NF-κB activity could be associated with a decrease in GLT-1 gene transcription. It has been observed in multiple cell types that DEX can inhibit NF-κB activation by enhancing the cellular levels of IκB-α [27,28] or the protein-protein interaction between activated GR and the p65 NF-κB subunit [29,30]. Thus, it is reasonable to speculate that DEX may act indirectly by inhibiting NF-κB activity and subsequently decreasing GLT-1 transcription. However, we could not exclude the possibility that DEX may inhibit transcriptional activation of GLT-1 through a direct DNA binding of activated GR to a specific site in the GLT-1 promoter. Further studies are required to test these possibilities.

We observed that DEX treatment decreases the expression of GLT-1 mRNA and protein in C6 glioma cells *in vitro*, consistent with our finding *in vivo* showing the reduction in GLT-1 expression in the frontal cortex by neonatal DEX treatment. However, this finding is in contrast with observations made in a previous study, which reported that DEX provokes an increase of GLT-1 transcription and protein levels in cortical astrocytes [31]. The reason for this seemingly contradictory finding is unclear but may be related to differences in experimental design as well as differences in cell types. Zschocke et al. [31] used rat primary cortical astrocytes and examined the extent of GLT-1 induction 72 hours after DEX (100 nM) treatment, whereas the present study examined the expression of GLT-1 in rat C6 glioma cells that were treated with DEX (100 μM) for 24–48 hours. Interestingly, as opposed to GLT-1, expression levels of GLAST mRNA and protein GLAST were not altered by DEX treatment in C6 glioma cells. These observations suggest that these two glial glutamate transporter subtypes have different sensitivities to glucocorticoid treatment. Further studies using different dosages of DEX are required to address this issue.

Because glucocorticoid therapy significantly reduces the incidence of CLD [17], it is difficult to avoid its use during neonatal life in preterm infants. There are two ways to reduce its adverse effects. One way is by lowing the DEX dosage or decreasing the duration of treatment and another way is in combination with other drugs. In the present study, our data revealed the potential clinical benefit of ceftriaxone to ameliorate DEX-induced potentiation of HI-induced brain injury. These results are in agreement with recent studies that ceftriaxone can offer neuroprotection in both *in vitro* and *in vivo* models of ischemic injury and motor neuron degeneration by preventing glutamate excitotoxicity [22,32]. Recent reports have also established that ceftriaxone may exert its neuroprotective effects by inducing GLT-1 transcription through increasing NF-κB binding to the GLT-1 promoter [25,33]. Although ceftriaxone has FDA approved for use in pediatric bacterial meningitis for a long time, there was no evidence of long-term neurodevelopmental sequelae of ceftriaxone treatment in the neonate. Our findings with ceftriaxone suggest that adjunct neuroprotective therapies that elevate GLT-1 activity may minimize glutamate excitotoxicity, thereby allowing a choice of DEX for use in neonates. Further large trials in humans are needed to confirm these results.

Perinatal HI-induced brain injury is one of major causes of CP. There is increasing evidence showing that early DEX administration in preterm infants may increase the incidence of CP [4-6]. In accordance with these clinical findings, the current results show that early DEX exposure is able to increase the vulnerability of the neonatal brain to subsequent HI damage. Although there are some studies indicating that DEX pretreatment can protect neonatal brain against subsequent HI injury [15,16,34], our results do not support a neuroprotective role for neonatal DEX treatment in cerebral HI. One possible explanation of these seemingly discrepant observations is the different doses and regimens of DEX used among studies. These findings reinforce the long-held view that the concentration and duration of glucocorticoid treatment are major factors determining the beneficial or detrimental effects of glucocorticoids in the brain [35]. In our model, DEX was administered over a long period (P1-3) in tapering doses in an attempt to mimic the longer treatment regimens commonly used in the neonatal intensive care setting [8,9,14,17,18]. While much caution is required when extrapolating data from animal models to the human condition, our findings highlight the risk for heightened developing brain vulnerability to HI injury associated with neonatal DEX treatment.

## Conclusion

In conclusion, our data provide evidence for a deleterious impact of neonatal DEX treatment on HI injury in the developing brain. Pretreatment wit ceftriaxone, perhaps due to an increase in GLT-1 expression, is able to stimulate glutamate uptake and overcome the excessive HI-induced brain injury resulting from neonatal DEX treatment. Although further investigations are needed to elucidate the molecular mechanisms involved in the GR-mediated downregulation of GLT-1 transcription, our findings demonstrate that supporting GLT-1 expression may exert beneficial effects to ameliorate the lasting enhancing effect of neonatal DEX treatment on glutamate-mediated excitotoxicity. These findings are of clinical importance because it is now difficult to avoid the use of corticosteroids in neonatology and perinatology to fight the problems of CLD.

## Methods

### Animals

Pregnant Sprague–Dawley rats (body weight 250–280 g) were single-housed under controlled illumination (12/12-hour light–dark cycle) and ambient temperature (24°C), and had *ad libitum* access to food and water. Pups were born on days 22–23 of gestation. On the day of birth (designated day 0), pups were removed from the nests and eight healthy pups (four males and four females) were randomly placed back with each dam. All experimental procedures were performed according to the National Institutes of Health Guide for the Care and Use of Laboratory Animals and were approved by the Institutional Animal Care and Use Committee of National Cheng Kung University.

### Cell culture

Rat C6 glioma cells were obtained from American Type Culture Collection (Manassas, VA) and cultured essentially as described by Amberger et al. [36]. Cells were cultured in 6 cm dishes in Dulbecco’s modified Eagle medium (DMEM; Invitrogen, San Diego, CA) supplemented with 10% fetal bovine serum (Invitrogen, Gaithersburg, MD), 2 mM L-glutamine, and penicillin (100 U/ml)/streptomycin (100 μg/ml) and incubated in 5% CO_2_-air humidified atmosphere at 37°C. Thereafter, half of the growth medium was replaced every three days. After cells reached ∼ 80% confluence, vehicle (0.1% DMSO) or DEX (100 μM) was added to the medium, and the plates were returned to the incubator until the assay was performed. The dose and duration of DEX treatment were selected on the basis of our pilot studies.

### Dexamethasone treatment *in vivo*

Each litter was assigned to two treatment groups: a SAL-treated and DEX-treated group. All pups within each litter were removed from their home cage and separated from their mother for injection and body weight measurement (between 11:00 and 13:00) for a period of 5 minutes. Only male offspring were used for experiments. Pups in the DEX group received a daily intraperitoneal injection of DEX (Sigma-Aldrich, St. Louis, MO) from P1 to P3. DEX was given in tapering doses of 0.5 mg/kg on P1, 0.3 mg/kg on P2, and 0.1 mg/kg on P3. Animals in the vehicle group received equivalent volumes of intraperitoneal injection of sterile SAL as the DEX-treated group. In some experiments, RU 38486 (40 mg/kg; Tocris Cookson Ltd., Bristol, UK) or ceftriaxone (200 mg/kg; Sigma-Aldrich) was administered intraperitoneally 1 hour before daily DEX application. Doses of RU 38486 and ceftriaxone were selected on the basis of previously published and our pilot studies [21,37]. Animals in the vehicle group received equivalent volumes of intraperitoneal injection of propylene glycol.

### Production of cerebral hypoxia-ischemia (HI)

Cerebral HI was produced as described previously [34]. Briefly, rat pups at P7 were anesthetized with halothane and underwent the right common carotid artery ligation through a longitudinal midline neck incision. The incision site was infiltrated with 2% lidocaine and the surgery lasted less than 5 minutes. The rat pups were returned to home cage with their dam for 3 hours followed by exposure to hypoxia (8% oxygen/92% nitrogen at 37°C) for 2 hours in a temperature-controlled plastic chamber. Sham animals underwent anesthesia and neck incision, the carotid artery was exposed without the ligation and was exposed to normoxic condition. The pups were returned to their dam after the hypoxic exposure.

### Assessment of infarct volume

Twenty-four hours after HI, rat pups were deeply anesthetized with isoflurane. The brains were removed carefully and dissected into coronal 2 mm sections using Leica VT1200S vibrating blade microtome (Leica, Nussloch, Germany). The slices were incubated in 2% TTC solution for 5 minutes in the dark, washed in phosphate buffered saline, and fixed in 10% formaldehyde. The infarct volume was traced and analyzed with Image J Software. The total infarct volume for each brain was calculated by summation of the infarcted area of all brain slices.

### Histochemical analysis

Twenty-four hours after HI, rat pups were deeply anesthetized with isoflurane and perfused transcardially with 0.1 M phosphate buffered saline (PBS) and 4% paraformaldehyde. After the perfusion, brains were removed and continue to fix in 4% paraformaldehyde for 48 hours at 4°C and then transferred to the solution containing 30% sucrose that immersed in 4°C for at least 48 hours before slicing. Coronal brain sections (25 μm) were collected, washed with 0.3% Triton X-100, and then incubated for blocking with solution containing 3% goat serum in PBS. The sections were mounted directly on gelatin-coated glass slides and dried. The slides were stained with 1.0% cresyl violet, dehydrated through a series of ethanol, cleared, and coverslipped with permount (Fisher Scientific, Electron Microscopy Sciences, Washington, PA). Stained sections were then examined under a computer-assisted Olympus BX51 microscope and images were taken with an Olympus DP70 microscope digital camera (Olympus, Tokyo, Japan).

### TUNEL analysis

Twenty-four hours after HI, rat pups were deeply anesthetized with isoflurane and Coronal brain sections (10 μm) were prepared as described above. The presence of apoptotic cells in the frontal cortex was detected by fluorometric detection of DNA fragmentation using an ApopTag® Fluorescein In Situ Apoptosis Detection Kit (S7110, Millipore, Bedford, MA) according to the manufacturer instructions. Slices were mounted using Vectashield mounting medium containing 4^′^,6-diamidino-2-phenylindole dilactate (DAPI) nuclear stain (Vector Laboratories, Burlingame, CA).

### Preparation of gliosomes and glutamate uptake assay

The gliosomal fractions of the frontal cortex were prepared as previously described with some modifications [38]. In brief, the microdissected tissue samples were homogenized in 0.32 M sucrose, 1 mM EDTA, 4 mM Tris and 10 mM glucose, pH 7.4, using a glass-Teflon homogenizer. Homogenates were centrifuged at 1,000 × g for 5 minutes, 4°C. The resultant pellet was discarded, and the supernatant was spun at 14,000 × g for 10 minutes in a microcentrifuge, 4°C. The pellets constituted the crude gliosomal fractions. The crude gliosomal fractions were resuspended in Krebs-Ringer buffer (in mM: 120 NaCl, 4.7 KCl, 2.2 CaCl_2_, 1.2 MgCl_2_, 25 HEPES, 1.2 MgSO_4_, 1.2 KH_2_PO_4_ and 10 glucose, pH 7.4) to give a protein concentration of 0.5 mg/ml that was determined by using a Pierce BCA Protein Assay Kit (Thermo Scientific, Rockford, IL). Glutamate uptake assay was performed as described previously [39]. Glutamate uptake in gliosomes was initiated by adding ^3^H-glutamate (10 nM, 20–60 Ci/mmol; Perkin Elmer, Boston, MA) and 30 μM unlabeled glutamate to the reaction tubes in a final volume of 500 μl of HEPES buffer solution. After incubation at 37°C for 5 minutes, the uptake was terminated by rapid filtration on glass-fiber filters using a tissue harvester under vacuum, and the filter was washed five times with ice cold HEPES buffer solution. Filters were dried overnight and countered on a liquid scintillation counter (Beckman Instruments, Fullerton, CA). Nonspecific uptake was determined with sodium-free solution that was prepared by replacing NaCl with choline chloride. The non-glutamate transporter-1 (non-GLT-1)-mediated glutamate uptake was calculated in the presence of GLT-1 inhibitor dihydrokainate (100 μM; Sigma-Aldrich).

### Western blotting

The microdissected tissue samples from the frontal cortex, the hippocampus or the striatum were transferred into ice-cold Tris–HCl buffer solution (TBS; pH 7.4) containing a cocktail of protein phosphatase and proteinase inhibitors (50 mM Tris–HCl, 100 mM NaCl, 15 mM sodium pyrophosphate, 50 mM sodium fluoride, 1 mM sodium orthovanadate, 5 mM EGTA, 5 mM EDTA, 1 mM phenylmethylsulfonyl fluoride, 1 μM microcystin-LR, 1 μM okadaic acid, 0.5% Triton X-100, 2 mM benzamidine, 60 μg/ml aprotinin, and 60 μg/ml leupeptin) to avoid dephosphorylation and degradation of proteins, and ground with a pellet pestle (Kontes glassware, Vineland, NJ). In some experiments, cultured C6 glioma cells were dissolved in ice-cold TBS containing a cocktail of protein phosphatase and proteinase inhibitors and collected by cell scraper. Samples were sonicated and spun down at 15,000 × g at 4°C for 10 minutes. The supernatant was then assayed for total protein concentration using Bio-Rad Bradford Protein Assay Kit (Hercules, CA). Each sample from tissue homogenate was separated using 8-10% SDS-PAGE gel. Following the transfer on nitrocellulose or polyvinylidene fluoride membranes, blots were blocked in buffer solution containing 5% milk and 0.1% Tween-20 in PBS (in mM: 124 NaCl, 4 KCl, 10 Na_2_HPO_4_ and 10 KH_2_PO_4_; pH 7.2) for 1 hour and then blotted for 2 hours at room temperature with antibodies that recognize GLT-1 (1:1000; Abcam Cambridge, MA), GLAST (1:1000; Abcam). NR2A (1:1000; Santa Cruz Biotechnology, Santa Cruz, CA), NR2B (1:1000; Santa Cruz Biotechnology) or β-actin (1:4000; Sigma-Aldrich, St Louis, MO). It was then probed with HRP-conjugated secondary antibody for 1 hour and developed using the ECL immunoblotting detection system (Amersham Biosciences, Buckinghamshire, UK), according to manufacturer’s instructions. Immunoblots were analyzed by densitometry using Bio-profil BioLight PC software (Vulber Lourmat, France). Only film exposures that were not saturated were used for quantification analysis. Expression of GLT-1, GLAST, NR2A or NR2B was evaluated relative to that for β-actin. Background correction values were subtracted from each lane to minimize the variability across membranes.

### Quantitative real-time RT-PCR

Total RNA was isolated from rACC tissue samples using a Tri Reagent kit (Molecular Research Center, Cincinnati, OH) and treated with RNase-free DNase (RQ1; Promega, Madison, WI) to remove potential contamination by genomic DNA. Total RNA (1 μg) from samples was reverse transcribed using a SuperScript cDNA synthesis kit (Invitrogen, Carlsbad, CA). Real-time PCR was performed on the Roche LightCycler instrument (Roche Diagnostics, Indianapolis, IN) using the FastStart DNA Master SYBR Green I kit (Roche Applied Science) according to the manufacturer’s instructions. The following primers were used: GLT-1 (1618–1780), 5^′^-ATTGACTCCCAACACCG-3^′^ (forward) and 5^′^-CATTGGCCGCCAGAGTTA-3^′^ (reverse); GLAST, 5^′^-TATACAGTGACAGTCATCGTC-3^′^ (forward) and 5^′^-ACAAATCTGGTGATGCGT-3^′^ (reverse); excitatory amino acid carrier 1 (EAAC1), 5^′^- GTCATTCTGCCACTGATTAT-3^′^ (forward) and 5^′^-GATGCCGTCTG.

AGTACAG-3^′^ (reverse); β-actin, 5^′^-TTCTACAATGAGCTGCGTGTGGC-3^′^ (forward) and 5^′^-CTCATAGCTCTTCTCCAGGGAGGA-3^′^ (reverse). PCR cycles consisted of an initial denaturation step at 95°C for 10 minutes, followed by 45 cycles of 10 seconds at 95°C, 10 seconds at 65°C, and 20 seconds at 72°C. After amplification, equal volumes of PCR products were subjected to electrophoresis on 1.5% (w/v) agarose gels and visualized with ethidium bromide. A melting curve was created at the end of the PCR cycle to confirm that a single product had been amplified. Data were analyzed by LightCycler quantification software to determine the threshold cycle above background for each reaction. The relative transcript amount of the gene of interest, which was calculated using standard curves of serial RNA dilutions, was normalized to that of β-actin of the same RNA.

### Data analysis

All data are expressed as means ± S.E.M. Number of animals used is indicated by n. The significance of the difference between the groups was calculated by one-way analysis of variance followed by Fisher’s least significant difference post hoc test. Probability values (*P*) of less than 0.05 were considered to represent significant differences.

## Abbreviations

CEF: Ceftriaxone; CP: Cerebral palsy; CLD: Chronic lung disease; DAPI: 4^′^,6-diamidino-2-phenylindole dilactate; DEX: Dexamethasone; GR: Glucocorticoid receptor; GLAST: Glutamate-aspartate transporter; GLT-1: Glutamate transporter-1; HI: Hypoxia-ischemia; NMDA: *N*-methyl-D-aspartate; P1-3: Postnatal days 1-3; PBS: Phosphate buffered saline; SAL: Saline; TTC: 2,3,5-triphenyltetrazolium chloride; Veh: Vehicle.

## Competing interests

The authors declare no competing financial interests.

## Authors’ contributions

KHC, CMY and CYY performed the experiments and the statistical analysis. KHC, CCH and KSH designed the study and wrote the manuscript. All authors read and approved the final manuscript.
